# Sex differences in acute and long‐term brain recovery after concussion

**DOI:** 10.1002/hbm.25591

**Published:** 2021-10-12

**Authors:** Nathan W. Churchill, Michael G. Hutchison, Simon J. Graham, Tom A. Schweizer

**Affiliations:** ^1^ Keenan Research Centre for Biomedical Science of St. Michael's Hospital Toronto ON Canada; ^2^ Neuroscience Research Program St. Michael's Hospital Toronto ON Canada; ^3^ Faculty of Kinesiology and Physical Education, University of Toronto Toronto ON Canada; ^4^ Department of Medical Biophysics University of Toronto Toronto ON Canada; ^5^ Physical Sciences Platform Sunnybrook Research Institute, Sunnybrook Health Sciences Centre Toronto ON Canada; ^6^ Faculty of Medicine (Neurosurgery), University of Toronto Toronto ON Canada; ^7^ The Institute of Biomaterials and Biomedical Engineering (IBBME) at the University of Toronto Toronto ON Canada

**Keywords:** ASL, brain injury, concussion, DTI, sex differences

## Abstract

Concussion is associated with acute disturbances in brain function and behavior, with potential long‐term effects on brain health. However, it is presently unclear whether there are sex differences in acute and long‐term brain recovery. In this study, magnetic resonance imaging (MRI) was used to scan 61 participants with sport‐related concussion (30 male, 31 female) longitudinally at acute injury, medical clearance to return to play (RTP), and 1‐year post‐RTP. A large cohort of 167 controls (80 male, 87 female) was also imaged. Each MRI session assessed cerebral blood flow (CBF), along with white matter fractional anisotropy (FA) and mean diffusivity (MD). For concussed athletes, the parameters were converted to difference scores relative to matched control subgroups, and partial least squares modeled the main and sex‐specific effects of concussion. Although male and female athletes did not differ in acute symptoms or time to RTP , all MRI measures showed significant sex differences during recovery. Males had greater reductions in occipital‐parietal CBF (mean difference and 95%CI: 9.97 ml/100 g/min, [4.84, 15.12] ml/100 g/min, *z* = 3.73) and increases in callosal MD (9.07 × 10^−5^, [−14.14, −3.60] × 10^−5^, *z* = −3.46), with greatest effects at 1‐year post‐RTP. In contrast, females had greater reductions in FA of the corona radiata (16.50 × 10^−3^, [−22.38, −11.08] × 10^−3^, *z* = −5.60), with greatest effects at RTP. These findings provide new insights into how the brain recovers after a concussion, showing sex differences in both the acute and chronic phases of injury.

## Graphical abstract

This study examined whether there are sex differences in acute and long‐term brain changes after concussion. Male athletes showed greater declines in cerebral blood flow, whereas both sexes showed unique alterations in white matter microstructure. These sex differences were present at acute injury and remained up to 1 year after medical clearance.

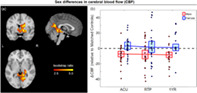

## 1 INTRODUCTION

Concussion is a highly prevalent form of mild traumatic brain injury (TBI) and a major health concern in sport and recreation, with approximately 1.6–3.8 million cases each year (Langlois, Rutland‐Brown, & Wald, 2006). It is associated with acute behavioral disturbances in the absence of overt neuroanatomical lesions, caused by microstructural injury and impaired physiological functioning (Giza & Hovda, 2014). There is also accumulating evidence that alterations in brain physiology persist well beyond the clinical determination of return to play (RTP) (Kamins et al., 2017). It is therefore critical to characterize the acute and long‐term patterns of brain recovery after concussion, to better inform guidelines of safe RTP progression in terms of potential vulnerability to reinjury (Guskiewicz et al., 2003; Vagnozzi et al., 2007), and to understand the mechanisms that may give rise to neurological sequelae later in life (Gavett, Stern, Cantu, Nowinski, & McKee, 2010; Guskiewicz et al., 2005).

It is also presently unclear to what extent demographic risk factors, such as age, sex, prior concussion history and preinjury mental health, influence physiological recovery and its relationship with RTP (Iverson et al., 2017). Among these factors, sex differences pose a particular conundrum. In the clinical domain, female athletes tend to be at greater risk for concussion, with more severe post‐concussion symptoms and neurocognitive disturbances, along with a greater risk for prolonged recovery (Covassin, Savage, Bretzin, & Fox, 2018; Merritt, Padgett, & Jak, 2019). By contrast, animal model studies of TBI often report reduced signs of pathophysiology and behavioral disturbances in females, suggesting a protective advantage (Rubin & Lipton, 2019). This apparent inconsistency between clinical outcomes and animal models of injury may be due to numerous factors, including sex differences in sport‐related injury biomechanics and willingness to disclose post‐concussion symptoms (Dick, 2009; Kroshus, Baugh, Stein, Austin, & Calzo, 2017). To better understand the complex factors contributing to sex differences in outcome after sport‐related concussion, neuroimaging may be used to directly measure the acute and long‐term changes in human brain physiology.

Two important indices of brain physiology are cerebral blood flow (CBF) and white matter (WM) microstructure, which have well‐established baseline differences between the sexes (Kodiweera, Alexander, Harezlak, McAllister, & Wu, 2016; Liu et al., 2012) and are highly sensitive to the effects of concussion, showing significant acute and chronic effects of injury (Churchill et al., 2017; Churchill et al., 2017a). There have been studies of sex differences in these parameters after concussion, however, none have examined recovery in the sport context with respect to the clinical determination of RTP (Fakhran, Yaeger, Collins, & Alhilali, 2014; Hamer & Churchill, 2019; McGlade, Rogowska, & Yurgelun‐Todd, 2015). It therefore remains unclear whether concussed male and female athletes both show acute brain changes that persist beyond RTP, and whether they have distinct patterns of long‐term recovery that generalize across sports.

To address these knowledge gaps, the present study used magnetic resonance imaging (MRI) to assess the CBF and WM microstructure of concussed athletes at acute injury, RTP, and 1‐year post‐RTP, along with a large normative athletic control group, with data acquired from a mixed sample of contact and noncontact sports. It was predicted that females would have smaller post‐concussion CBF disturbances than males, given the observed neuroprotective effects of female sex hormones (Wagner et al., 2004), and their more reactive cerebrovascular system (Kastrup, Thomas, Hartmann, & Schabet, 1997; Tallon, Barker, Nowak‐Flück, Ainslie, & McManus, 2020) which may better compensate for post‐concussion CBF disturbances. Conversely, it was predicted that females would show greater post‐concussion alterations in WM tissue than males, based on evidence of reduced neck musculature among female athletes, decreasing their ability to buffer head impacts (Tierney, 2004; Vasavada, Danaraj, & Siegmund, 2008) and, combined with reduced axonal fiber volumes (Kodiweera et al., 2016), increasing their vulnerability to axonal injury. Furthermore, it was predicted that CBF and WM effects would be present at acute injury and persist beyond RTP, given prior evidence of persistent brain changes in this cohort (Churchill et al., 2017b; Churchill, Hutchison, Graham, & Schweizer, 2019a). These hypotheses were evaluated in a multivariate partial least squares (PLS) framework with cohort‐adjusted MRI data, to control for baseline differences due to sex and other demographic factors.

## 2 METHODS

### 2.1 Study participants

A total of 61 concussed athletes were recruited consecutively from multiple university‐level sport teams at a single institution through the academic sport medicine clinic (see Table 1 for athlete numbers by sport), following a concussion diagnosis. Diagnosis was determined by a staff physician following direct or indirect contact to the head with signs and/or symptoms per Concussion in Sport Group guidelines (McCrory et al., 2013), followed by standard neurologic assessment of cranial nerves, gait, balance, and gross motor function. Imaging was conducted at acute injury (ACU; 1–7 days post‐injury), at medical clearance to RTP and at 1‐year post‐RTP (1YR). Some of the concussed athletes had missed imaging sessions. The number of participants retained at each time point was ACU (53/61), RTP (51/61), and 1YR (32/61). Attrition was not significantly related to demographic variables (age, sex, concussion history) or clinical variables (symptom severity, time to RTP), based on Spearman correlations at a False Discovery Rate (FDR) threshold of 0.05. As a control group, 167 athletes without recent concussion were also consecutively recruited and imaged at the start of their competitive season. In terms of exclusion criteria, none of the athletes recruited for the study had a history of neurological or psychiatric diseases or sensory/motor impairments. Any athletes, concussed or control, with a prior history of concussion (HOC) were also required to be fully recovered clinically, based on graded exertional protocols with cognitive and symptom monitoring (McCrory et al., 2017). All athletes completed baseline assessments using the most recent Concussion in Sport Group's Sport Concussion Assessment Tool (SCAT3 or 5) before the beginning of their athletic seasons. Athletes diagnosed with concussion also completed SCAT assessments at acute injury and at RTP. The study was carried out in accordance with the Canadian Tri‐Council Policy Statement 2 and approval of the University of Toronto and St. Michael's Hospital research ethics boards, with all participants giving free and written informed consent.

**TABLE 1:** Demographic and symptom data for male and female athletes with concussion and controls

	Male control	Female control	Male concussed	Female concussed
Age (years)	20.7 ± 2.3	19.8 ± 1.6	20.8 ± 2.0	19.9 ± 1.9
History of concussion (HOC)	36/80 (45%)	37/87 (43%)	19/30 (63%)	17/31 (55%)
^a^Number of prior concussions	2 [1, 2]	2 [1, 2]	2 [1, 2]	2 [1, 2]
^a^Months since last concussion	37 [18, 59]	24 [12, 36]	39 [16, 74]	24 [10, 36]
Sport	Volleyball (13) Hockey (21) Soccer (15) Football (11) Rugby (5) Basketball (3) Lacrosse (10) Water polo (1) Squash (1) –	Volleyball (21) Hockey (30) Soccer (10) – Rugby (11) Basketball (10) Lacrosse (5) – – –	Volleyball (3) Hockey (7) – Football (7) Rugby (7) Basketball (2) Lacrosse (3) Water polo (1) – –	Volleyball (3) Hockey (6) Soccer (1) – Rugby (14) Basketball (2) Lacrosse (3) Water polo (1) – Mountain biking (1)
Days to RTP	–	–	27 [16, 65]	44 [13, 85]
Total symptoms (baseline)	2 [0, 5]	2 [0, 4]	3 [1, 5]	4 [2, 8]
Total symptoms (ACU)	–	–	9 [4, 17]	10 [6, 16]
Total symptoms (RTP)	–	–	0 [0, 2]	1 [0, 2]
Total severity (baseline)	2 [0, 9]	3 [0, 6]	3, [1 8]	5 [2, 11]
Total severity (ACU)	–	–	13 [4, 27]	16 [7, 41]
Total severity (RTP)	–	–	0 [0, 2]	2 [0, 4]

*Notes*: Age is summarized by the mean and standard deviation. Number of prior concussions, months since last concussion, and symptom scores are summarized by the median and interquartile range. For symptom scores, values are reported at pre‐injury baseline, at acute injury (ACU), and at return to play (RTP).

^a^ Reported for the subset of athletes with history of concussion (HOC).

### 2.2 Magnetic resonance imaging

Athletes were imaged using a 3 Tesla MRI system (Magnetom Skyra) with a standard 20‐channel head coil. Structural imaging included: three‐dimensional T1‐weighted Magnetization Prepared Rapid Acquisition Gradient Echo imaging [MPRAGE: inversion time (TI)/echo time (TE)/repetition time (TR) = 1,090/3.55/2,300 ms, flip angle (*θ*) = 8°, 192 sagittal slices with field of view (FOV) = 240 × 240 mm, 256 × 256 pixel matrix, 0.9 mm slice thickness, 0.9 × 0.9 mm in‐plane resolution, with bandwidth (BW) = 200 Hz per pixel (Hz/px)], fluid‐attenuated inversion recovery imaging (FLAIR: TI/TE/TR = 1,800/387/5,000 ms, 160 sagittal slices with FOV = 230 × 230 mm, 512 × 512 matrix, 0.9 mm slice thickness, 0.4 × 0.4 mm in‐plane resolution, BW = 751 Hz/px), and susceptibility‐weighted imaging (SWI: TE/TR = 20/28 ms, *θ* = 15°, 112 axial slices with FOV = 193 × 220 mm, 336 × 384 matrix, 1.2 mm slice thickness, 0.6 × 0.6 mm in‐plane resolution, BW = 120 Hz/px). The structural scans were inspected by an MRI technologist during imaging and later reviewed by a neuroradiologist, with clinical reporting if abnormalities were identified. Statistical testing was also performed by obtaining mean, variance, and skew of voxel signal intensity distributions for masked MPRAGE, FLAIR, and SWI images, generating a *Z*‐score for per imaging sequence and athlete relative to the control distribution and identifying significant outliers at *p* < .05 (Bonferroni‐adjusted). No abnormalities (WM hyperintensities, contusions, micro‐hemorrhage, or statistical outliers) were detected for the concussed athletes or controls.

#### 2.2.1 Arterial spin labeling

2D pulsed arterial spin labeling (ASL) was acquired using the PICORE QUIPSS II sequence (TE/TR = 12/2,500 ms, TI1/TI1s/TI2 = 700/1,600/1,800 ms, *θ* = 90°, 14 oblique‐axial slices with FOV = 256 × 256 mm, 64 × 64 matrix, 8.0 mm slice thickness with 2.0 mm gap, 4.0 × 4.0 mm in‐plane resolution, BW = 2,368 Hz/px). A single calibration image was acquired to estimate the equilibrium magnetization *M*_0_, together with a series of 45 tag‐control image pairs. Data were processed and analyzed using Analysis of Functional Neuroimages (AFNI) software (afni.nimh.nih.gov) and customized algorithms. Rigid‐body motion correction of tag‐control image pairs was performed using *3dvolreg* to align to the *M*_0_ image. Filtering of outlier tag‐control pairs was performed using an established protocol (Tan et al., 2009), followed by spatial smoothing with *3dmerge*, using a 3D Gaussian kernel with 6 mm isotropic full width at half‐maximum (FWHM). Voxel‐wise estimates of CBF were calculated in units of ml/100 g/min based on the mean difference of all tag‐control pairs, using established kinetic modeling parameters (Churchill et al., 2017a). The CBF maps were then co‐registered using the FMRIB Software Library (FSL; https://fsl.fmrib.ox.ac.uk). The MNI152 template was used as reference and *flirt* was used to compute the rigid‐body alignment of each participant's mean ASL volume to their T1 image, along with the affine alignment of the T1 image to the template. The net affine transform was then applied to the ASL data and images were resampled at 3 mm isotropic resolution. To ensure that only gray matter (GM) regions were analyzed, voxels were retained that intersected with the MNI152 brain mask and a GM mask. The latter was obtained by applying *fast* to all participant T1 images, producing segmented GM, WM, and cerebrospinal fluid (CSF) maps. The maps were then aligned to the MNI152 template using *fslvbm* and smoothed with a 3D Gaussian kernel with 6 mm isotropic FWHM, followed by group averaging. A GM mask was chosen to include only regions with probability *p*(GM) > *p*(WM) + *p*(CSF). Remaining voxels that overlapped with ventricles of the template were afterwards removed manually. To further control against WM partial volume effects, an additional masking step was performed, by retaining only voxels with mean control CBF values >20 ml/100 g/min.

#### 2.2.2 Diffusion tensor imaging

A diffusion tensor imaging (DTI) protocol was performed (66 axial slices with FOV = 240 × 240 mm, 120 × 120 matrix, 2.0 mm slice thickness, 2.0 × 2.0 in‐plane resolution, BW = 1,736 Hz/Px), consisting of 30 diffusion‐weighting directions (TE/TR = 83/7,800 ms, b = 700 s/mm^2^, with nine b0 scans). The data were processed using FSL utilities and custom software. The *eddy* protocol was used to perform simultaneous correction of eddy currents and rigid‐body head motion, *bet* was used to mask out nonbrain voxels, and *dtifit* was used to calculate voxel‐wise measures of fractional anisotropy (FA) and mean diffusivity (MD). Co‐registration of DTI maps to a common template was obtained using Diffusion Tensor Imaging ToolKit (DTI‐TK) software with default parameter settings (dti-tk.sourceforge.net). The IXI Aging DTI Template 3.0 was used as an initial reference, and a randomly selected, demographically matched subgroup of 60 athletic controls was used to generate an athlete template [mean ± SD age: 20.2 ± 1.7 years, 31/60 female (52%), 26/60 with HOC (43%)]. For this group, a bootstrapped template was obtained with *dti_template_bootstrap*, affine alignment and template updating was done using *dti_affine_population* (three iterations), then diffeomorphic alignment and template updating was done with *dti_diffeomorphic_population* (six iterations). The transform from athletic template to MNI space was afterward obtained using the IIT Human Brain Atlas' mean tensor template, by sequentially applying rigid (*dti_rigid_reg*), affine (*dti_affine_reg*), and diffeomorphic (*dti_diffeomorphic_reg*) registration steps. For all athletes in this study, transforms to the athlete group template were then obtained by sequentially applying rigid (*dti_rigid_reg*), affine (*dti_affine_reg*), and diffeomorphic (*dti_diffeomorphic_reg*) registration steps. After, the net transforms into MNI space were computed using *dfRightComposeAffine* and were applied to DTI parameter maps via *deformationScalarVolume*. During registration, images were resampled to 3 × 3 × 3 mm resolution, and a 6 mm FWHM 3D Gaussian smoothing kernel was applied to reduce spatial noise (e.g., due to scanner noise, head motion, and minor alignment errors). Analysis was performed within a mask of WM regions where FA >0.30 in the group template, with manual segmentation and exclusion of brain stem areas, as they tend to exhibit substantial field inhomogeneity.

#### 2.2.3 Outlier detection

As the study focused on multivariate covariance relationships, which are sensitive to extreme data points, all imaging data were tested for outliers using a multivariate approach (see Appendix‐1 for details). For the CBF maps, two (2) control, two (2) RTP and two (2) 1YR scans were identified as outliers. For the FA maps, two (2) control scans were identified as outliers and, for the MD maps, two (2) control scans were identified. These outlier scans were then excluded from further analysis of concussion effects.

### 2.3 Clinical and demographic data: Sex and concussion recovery

Participant demographics are listed in Table 1 by sex, including age, HOC, features of HOC (total number of concussions and months since the last injury), along with time to RTP for concussed athletes. Clinical scores are also reported for SCAT symptoms. A total symptom severity score was obtained by summing across a 22‐item scale, with each item receiving a seven‐point Likert scale rating. A total symptom score was also obtained by counting the number of symptoms with nonzero ratings. For male and female athlete groups, scores were tested for differences relative to baseline, via nonparametric Wilcoxon paired‐measures tests. Subsequent analyses compared concussed male and female athletes in terms of demographic and clinical variables including age, rates of HOC, features of HOC, time to RTP, number of symptoms and symptom severity, using two‐sample Wilcoxon tests with significance assessed at an FDR of 0.05.

In addition, given mixed evidence of distinct symptom profiles for male and female athletes (Merritt et al., 2019), supplemental analyses examined symptom severity scores summed over specific domains at acute injury, including somatic (nine items: “headache”, “pressure in head,” “neck pain,” “nausea,” “dizziness,” “blurred vision,” “balance problems,” “sensitivity to light,” “sensitivity to noise”), cognitive (six items: “feeling slowed down,” “feeling ‘in a fog,’” “don't feel right,” “difficulty concentrating,” “difficulty remembering,” “confusion”), sleep‐related (three items: “fatigue,” “drowsiness,” and “trouble falling asleep”) and mood‐related (four items: “more emotional,” “irritability,” “sadness,” and “nervous/anxious”). For each of these domains, concussed male and female athlete groups were compared using two‐sample Wilcoxon tests, with significance determined at an FDR of 0.05.

### 2.4 Neuroimaging data: sex and concussion recovery

For each MRI parameter (CBF, FA, and MD), analyses were performed to identify (a) the main effects of concussion, common to both male and female athletes, and (b) the sex‐specific effects of concussion. Differences in the MRI parameter values of concussed males and females reflect a mixture of baseline sex differences in neurophysiology and differences in concussion response. To isolate the effects of concussion from those attributable to sex and other demographic factors, concussed athletes were analyzed after converting their MRI data into difference scores relative to normative controls, using the approach originally published in Churchill et al. (2019a). For each concussed athlete *s=1…61*, the voxel values $x_{s}$ were converted to difference scores as follows: all controls were identified that matched the concussed athlete on sex and presence or absence of HOC, with ≤2 years age difference; based on these criteria, a median of 29 controls (interquartile range: 27, 36) was matched to each concussed athlete. From the control subgroup, a robust mean $m_{\left(s\right)}$ was then calculated at each voxel, via location M‐estimator with a tuning parameter *k* = 1.35; the voxel difference scores $∆x_{s}=x_{s}-m_{\left(s\right)}$. This produced “difference maps” (ΔCBF, ΔFA, ΔMD) that robustly quantify how much each concussed athlete deviated from a demographically comparable cohort without recent injury. For a given concussed athlete, the same control subgroup was matched to each imaging session, to ensure a consistent baseline reference during longitudinal analysis.

For each MRI parameter, an uncentered task PLS analysis was then performed on (a) the maps of mean $∆x_{s}$ obtained for ACU, RTP, and 1YR, and (b) the maps of correlations between $∆x_{s}$ and sex (binary variable, 0 = male and 1 = female) obtained for ACU, RTP, and 1YR. The PLS approach produces paired components, including a “voxel salience” map, reflecting the pattern of brain regions that had greatest covariation across sessions, and a set of “session saliences”, reflecting how much each imaging session expressed this brain pattern. The first PLS component was reported for each MRI parameter, which explains the most data covariance overall. Repeated‐measures bootstrap resampling was used to perform inference on PLS saliences (1,000 iterations), with resampling units consisting of a concussed athlete's ACU, RTP, and 1YR difference maps. The standardized effect sizes for voxel and session saliences were obtained as bootstrap ratios (BSRs; mean/ standard error), along with normal *p*‐values. Significant brain regions were identified after adjusting for multiple comparisons by applying a voxel‐wise threshold of *p* = .005, followed by cluster‐size thresholding at an adjusted *p* = .05, using the AFNI program *3dFWHMx* to estimate the spatial smoothness of parameter maps, followed by *3dClustSim* to obtain the minimum cluster size threshold. To account for missing data, PLS model fitting was performed with missing data entries omitted from the cost function.

For the PLS analyses identifying significant concussion effects, the MRI parameter values were averaged over significant voxels and the mean $∆x_{s}$ values were reported, along with bootstrapped 95% confidence intervals (95% CIs), BSRs and *p*‐values, for each imaging session. For main effects analyses, statistics were calculated for all concussed athletes, whereas for analyses of sex effects, statistics were calculated separately for male and female concussed athlete groups. Significance testing across imaging sessions was conducted at an FDR of 0.05. To mitigate bias and efficiency loss due to missing data, multiple imputation was performed before calculating bootstrapped statistics, using the SOFT‐IMPUTE algorithm (Mazumder, Hastie, & Tibshirani, 2010). This technique was applied to the matrix of partially complete subject × time imaging data to impute missing values at each bootstrap iteration, with soft threshold *λ* chosen to minimize cross‐validated mean squared reconstruction error (200 iterations with 5% of data held out).

## 3 RESULTS

### 3.1 Clinical and demographic data: sex and concussion recovery

Demographic and clinical information are reported for the athlete cohorts in Table 1. For all cohorts, athletes were drawn from a mixture of different sports, although for both sexes, the predominant sport among controls was hockey. Similarly, concussed athletes were mainly from higher‐risk contact and collision sports for both sexes, including rugby, hockey, and football. For concussed athletes (male and female) the number and severity of symptoms were significantly elevated at acute injury relative to controls and their own baseline (*z* ≥ 4.82, *p* < .001, for all tests) at an FDR of 0.05 but were no longer elevated at RTP (*z* ≤ −3.24, *p* ≥ .999, for all tests). The female concussed group tended to be younger than their male counterparts (*z* = −1.98, *p* = .048), although the effect was nonsignificant at an FDR of 0.05, and the groups did not differ in HOC rates (*z* = −0.66, *p* = .510). In terms of HOC features, including number of concussions and time since the last injury, male and female concussed groups did not differ significantly from their respective control cohorts, nor did they differ significantly from each other (|*z*| ≤ 0.93, *p* ≥ .350, for all tests). Female athletes had a higher median number of days to RTP than males, but the difference was not significant (*z* = 0.25, *p* = .803). Similarly, female athletes had higher median symptom scores than males at baseline, acute injury, and RTP, but none of the differences were statistically significant (|*z*| < 1.86 and *p* ≥ .062, for all tests). Furthermore, supplemental analyses of somatic, cognitive, sleep‐related, and mood‐related symptom subdomains found no evidence of domain‐specific sex differences (|*z*| ≤ 1.25, *p* ≥ .210, for all tests). None of the concussed athletes in this study had acquired a new concussion between ACU and 1YR, and all had returned to normal school, work, social, and sport activities at this time.

### 3.2 Neuroimaging data: sex and concussion recovery

**FIGURE 1:**
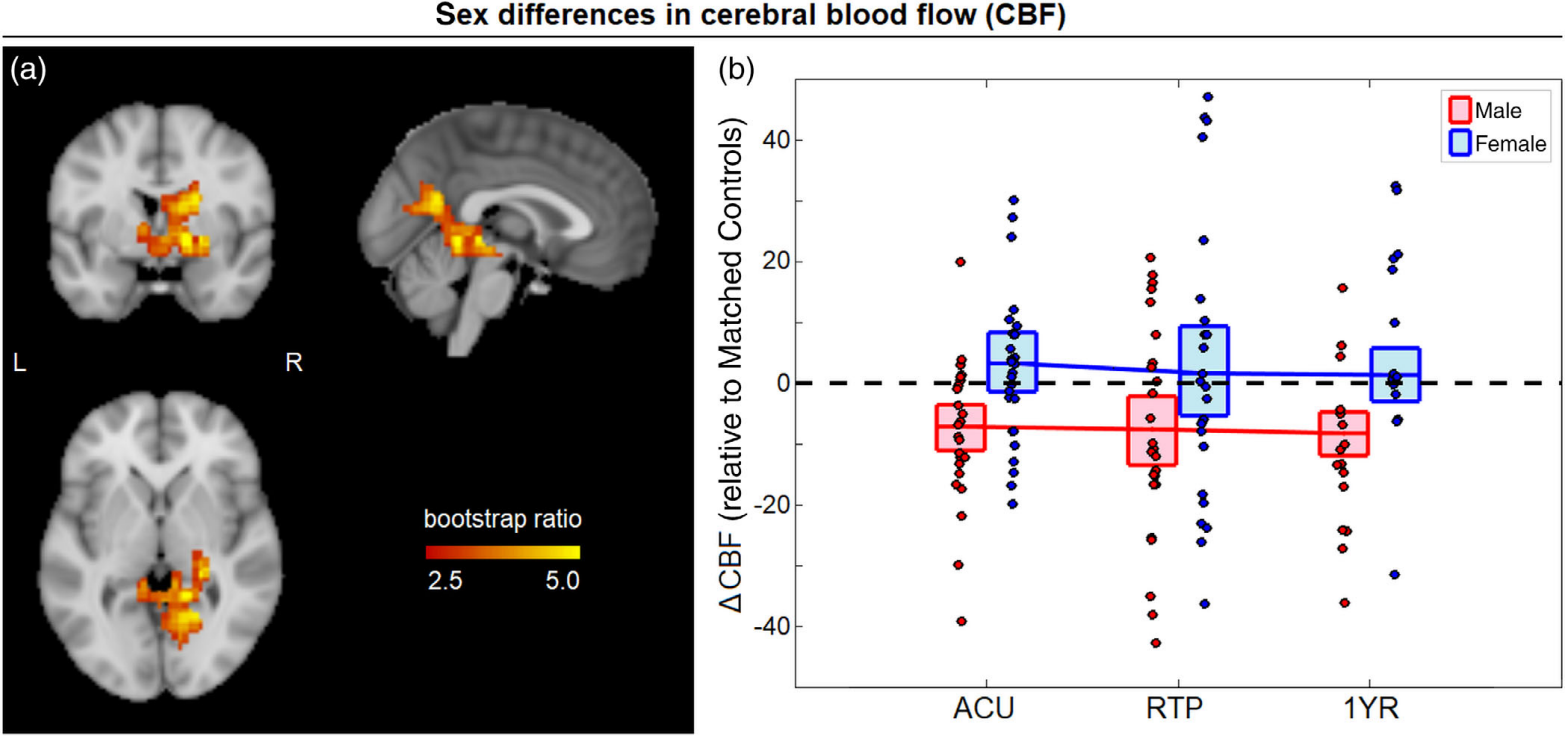
(a) Brain areas with significant sex differences in the effect of concussion on cerebral blood flow (CBF); maximum intensity projections are shown in orthogonal planes (MNI coordinates: *x* = 0, *y* = 0, *z* = 0). (b) The distribution of ΔCBF values for concussed male and female athletes (i.e., differences relative to matched controls) plotted for imaging sessions of acute injury (ACU), return to play (RTP), and 1‐year post‐RTP (1YR). The red/blue lines denote group means and boxes denote 95% confidence bounds on the means, with dashed black line indicating zero effect

Significant clusters are summarized for all neuroimaging analyses in Table 2 and statistics of effect, averaged over all clusters, are reported in Table 3. For CBF, a significant main effect of concussion was identified (69.3% of PLS covariance, 95%CI: [48.2%, 77.8%]) with frontal, temporal, and parietal clusters. In these regions, concussed athletes had reduced CBF relative to controls, with session salience effects that increased from ACU (BSR = −0.39; *p* = .697) to RTP (BSR = −2.44; *p* = .015) and 1YR (BSR = −11.15; *p* < .001), although only the latter two sessions were significant at an FDR of 0.05. A significant effect of sex on concussion response was also identified (58.9% of PLS covariance, 95%CI: [44.3%, 68.7%]) for a single cluster centered on the visual cortex and extending into the cuneus and precuneus (Figure 1). In these regions, there was a positive effect of sex on CBF, with significant session saliences at ACU (BSR = 2.59; *p* = .010), RTP (BSR = 2.58, *p* = .010) and 1YR (BSR = 4.96; *p* < .001). This corresponded to a more positive post‐concussion ΔCBF among female athletes (mean difference: 9.97 ml/100 g/min, [4.84, 15.12] ml/100 g/min, BSR = 3.73; *p* < .001, collapsing over significant imaging sessions). Examining the cohorts individually, male athletes had significantly reduced CBF relative to controls at all sessions. Female athletes, by contrast, had modest elevations in CBF relative to controls, with effects that were uniformly nonsignificant.

**TABLE 2:** Cluster report for main effects and sex differences in concussion response, for cerebral blood flow (CBF), fractional anisotropy (FA), and mean diffusivity (MD)

	Analysis	Cluster	Center of mass			Brain region	Cluster size (mm^3^)	Peak value (BSR)
ΔCBF	Main		39	39	6	Inferior frontal (triang. part) R	8,316	5.24
			3	33	30	Anterior cingulate R	7,209	5.18
			0	−81	−9	Calcarine L	4,428	5.81
			−51	−30	15	Superior temporal L	4,320	4.45
			−6	−48	39	Precuneus L	3,186	3.67
			33	27	42	Middle frontal R	2,538	3.92
			−3	−54	12	Precuneus L	1,539	3.78
			60	−36	24	Superior temporal R	1,512	4.00
			6	−81	36	Cuneus R	1,431	4.08
			51	−6	−6	Superior temporal R	1,296	3.52
			45	6	42	Precentral R	1,242	4.53
	Sex		12	−51	6	Lingual R	8,234	5.38
ΔFA	Main	–	–	–	–	–	–	–
	Sex		24	33	3	Anterior corona radiata R	1,620	4.83
			−21	12	36	Superior corona radiata L	1,323	4.13
			21	9	42	Superior corona radiata R	1,296	5.40
			−27	−42	24	Posterior corona radiata L	1,026	4.09
			−21	−27	48	Superior corona radiata L	999	4.59
			−24	27	3	Anterior corona radiata L	864	4.79
ΔMD			27	−12	42	Superior corona radiata R	35,721	6.25
			−24	−18	42	Superior corona radiata L	33,426	7.24
			3	−21	21	Body of corpus callosum R	3,510	4.82
			−24	−81	−3	Posterior thalamic radiation L	1,026	4.15
			36	−39	3	Posterior thalamic radiation R	756	4.06

*Notes*: The cluster centers of mass are in MNI coordinates and brain regions are identified based on the nearest labeled gray matter region in the automated anatomical labeling (AAL) atlas (CBF), or the nearest labeled white matter tract in the Johns Hopkins University (JHU) atlas (FA and MD). The peak value for each cluster is given in terms of bootstrap ratio (BSR).

**TABLE 3:** Statistics of effect, reflecting the mean deviation of concussed athlete values from normative values, for cerebral blood flow (CBF), fractional anisotropy (FA), and mean diffusivity (MD)

	Mean	95% CI	BSR	*p*‐value
ΔCBF (ml/100 g/min)				
All				
ACU	−0.49	[−2.81, 2.03]	−0.40	.682
RTP	−4.58	[−7.22, −2.09]	−3.53	.002*
1YR	−8.00	[−9.37, −6.58]	−11.31	<.001*
Male				
ACU	−7.19	[−11.12, −3.59]	−3.80	<.001*
RTP	−7.62	[−13.50, −2.19]	−2.64	.006*
1YR	−8.29	[−11.91, −4.78]	−4.36	<.001*
Female				
ACU	3.13	[−1.47, 8.13]	1.28	.198
RTP	1.60	[−5.38, 9.33]	0.40	.742
1YR	1.31	[−3.02, 5.74]	0.63	.544
ΔFA (×10^−3^)				
All				
ACU	–	–	–	–
RTP	–	–	–	–
1YR	–	–	–	–
Male				
ACU	10.56	[5.23, 15.96]	3.86	<.001*
RTP	10.89	[6.14, 16.46]	4.24	<.001*
1YR	8.94	[4.54, 13.50]	3.90	<.001*
Female				
ACU	−6.46	[−10.17, −2.67]	−3.30	<.001*
RTP	−7.71	[−11.95, −3.84]	−3.77	<.001*
1YR	−5.77	[−9.15, −2.74]	−3.48	<.001*
ΔMD (×10^−5^)				
All				
ACU	1.15	[0.62, 1.68]	4.29	<.001*
RTP	1.22	[0.70, 1.73]	4.44	<.001*
1YR	1.58	[1.25, 1.90]	9.26	<.001*
Male				
ACU	5.76	[2.19, 9.41]	3.07	.004*
RTP	5.01	[1.52, 8.49]	2.79	.002*
1YR	6.29	[2.65, 10.19]	3.26	<.001*
Female				
ACU	−3.08	[−6.85, 0.88]	−1.56	.134
RTP	−3.71	[−7.65, 0.37]	−1.85	.068
1YR	−3.09	[6.98, 0.71]	−1.61	.118

*Notes*: The “all” entries represent group estimates of effect, for both male and female athletes, averaged over main effect clusters in Table 2. The “male” and “female” entries represent sex‐specific estimates of effect, averaged over sex effect clusters in Table 2. Effects are reported at acute injury (ACU), return to play (RTP) and 1 year post‐RTP (1YR). Statistics include the mean, bootstrapped 95% confidence interval (95%CI), bootstrap ratio (BSR), and empirical *p*‐value. A “*” identifies significant imaging sessions at a False Discovery Rate threshold of 0.05.

**FIGURE 2:**
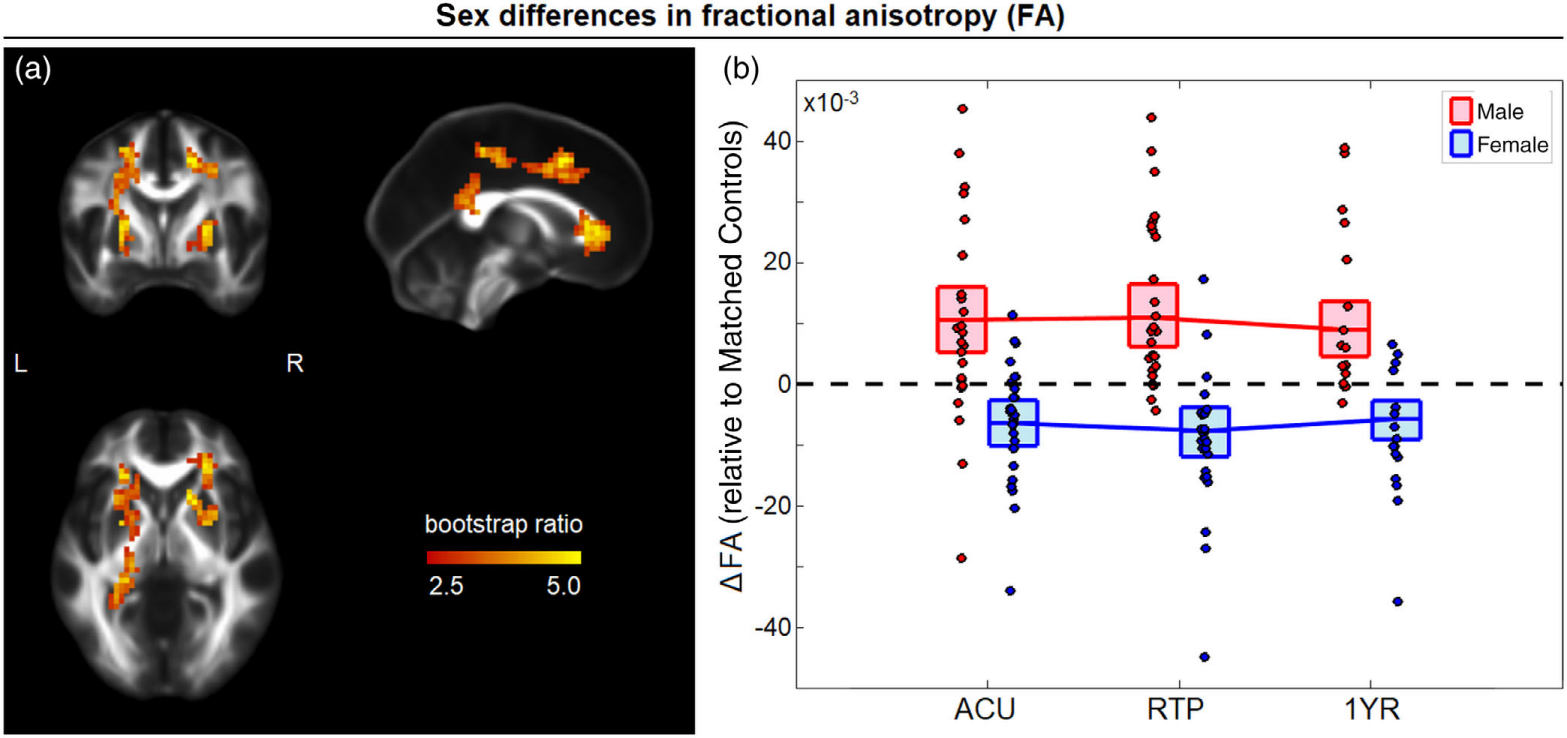
(a) Brain areas with significant sex differences in the effect of concussion on fractional anisotropy (FA); maximum intensity projections are shown in orthogonal planes (MNI coordinates: *x* = 0, *y* = 0, *z* = 0). (b) The distribution of ΔFA values for concussed male and female athletes (i.e., differences relative to matched controls) plotted for imaging sessions of acute injury (ACU), return to play (RTP), and 1‐year post‐RTP (1YR). The red/blue lines denote group means and boxes denote 95% confidence bounds on the means, with dashed black line indicating zero effect

For FA, no significant main effects of concussion were identified across the imaging sessions. However, a significant effect of sex on concussion response was identified (74.5% of PLS covariance, 95%CI: [62.9%, 81.2%]) for clusters throughout the corona radiata (Figure 2). In these regions, there was a negative effect of sex on FA, with significant session saliences at ACU (BSR = −7.96; *p* < .001), RTP (BSR = −8.38; *p* < .001), and 1YR (BSR = −7.41, *p* < .001). This corresponded to a more negative post‐concussion ΔFA among female athletes (mean difference: 16.50 × 10^−3^, [−22.38, −11.08] × 10^−3^, BSR = −5.60; *p* < .001, collapsing over significant imaging sessions). Examining the cohorts individually, male athletes had higher FA relative to controls at all sessions, with uniformly significant effects. Conversely, female athletes had reduced FA relative to controls at all imaging sessions, also with uniformly significant effects that were greatest at RTP.

**FIGURE 3:**
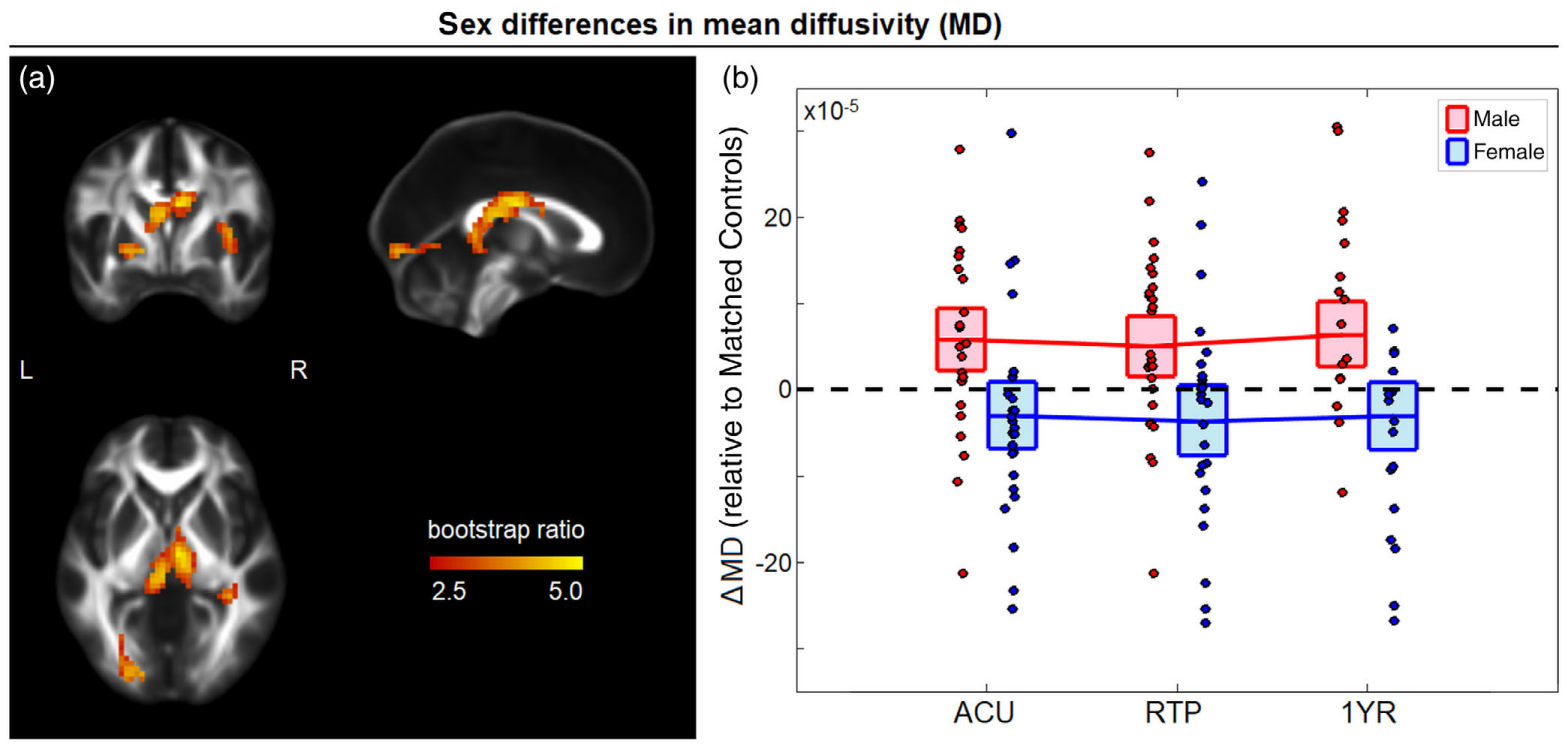
(a) Brain areas with significant sex differences in the effect of concussion on mean diffusivity (MD); maximum intensity projections are shown in orthogonal planes (MNI coordinates: *x* = 0, *y* = 0, *z* = 0). (b) The distribution of ΔMD values for concussed male and female athletes (i.e., differences relative to matched controls) plotted for imaging sessions of acute injury (ACU), return to play (RTP), and 1‐year post‐RTP (1YR). The red/blue lines denote group means and boxes denote 95% confidence bounds on the means, with dashed black line indicating zero effect

For MD, a significant main effect of concussion was identified (86.3% of PLS covariance, 95%CI: [67.4%, 88.7%]), with large clusters centered on the superior corona radiata, but extending into the anterior corona radiata and superior longitudinal fasciculi. In these regions, concussed athletes had elevated MD relative to controls, with session salience effects that increased from ACU (BSR = 5.25; *p* = <.001) to RTP (BSR = 6.20; *p* = <.001) and 1YR (BSR = 7.90; *p* < .001) and were uniformly significant at an FDR of 0.05. In addition, a significant effect of sex on concussion response was identified (74.0% of PLS covariance, 95%CI: [54.5%, 82.6%]) for clusters in the body of the corpus callosum and the posterior thalamic radiation (Figure 3). In these regions, there was a negative effect of sex on FA, with significant session saliences at ACU (BSR = −3.89; *p* < .001), RTP (BSR = −4.19; *p* < .001) and 1YR (BSR = −6.99; *p* < .001). This corresponded to a more negative post‐concussion ΔMD among female athletes (mean difference: 9.07 × 10^−5^, [−14.14, −3.60] × 10^−5^, BSR = −3.46; *p* < .001, collapsing over significant imaging sessions). Examining the cohorts individually, male athletes had elevated MD relative to controls at all sessions, with uniformly significant effects. Female athletes showed slightly reduced MD at all imaging sessions, but the effects were uniformly nonsignificant.

## 4 DISCUSSION

There is a growing body of research examining brain physiology after sport‐related concussion, with evidence that recovery extends beyond medical clearance to RTP. To date, however, differences in the long‐term recovery of male and female athletes have received more limited research attention. This study provides the first evidence of sex‐specific concussion effects that are present at acute injury and last up to 1‐year post‐RTP. The results were obtained using a novel approach, in which multivariate PLS analysis was applied to cohort‐adjusted MRI difference maps. The study findings partly support our initial hypotheses, as female athletes showed smaller CBF disturbances than male athletes, but both sexes showed distinct patterns of altered WM microstructure. In both groups, the CBF and WM effects also persisted beyond the clinical determination of RTP.

Importantly, sex differences in MRI measures occurred in the absence of significant differences in clinical presentation. The examined measures include time to RTP, total number and severity of symptoms, along with assessments of specific symptom domains. This may be contrasted with surveys of the clinical literature, in which female athletes tend to have longer recovery times and more severe post‐concussion symptoms than their male counterparts (Covassin et al., 2018; Merritt et al., 2019). However, there remains substantial variability in the effect sizes reported across studies, and efforts to aggregate findings across multiple sports have reported more limited effects (Abrahams, Mc Fie, Patricios, Posthumus, & September, 2014; Cheng et al., 2019), which is consistent with the present multisport investigation. Given the significant MRI results, which identified sex differences in neurobiology across multiple sports, it is presently unclear whether the absence of corresponding differences in clinical outcome is due to MRI findings being unrelated to symptom presentation, or whether the relationships are obscured by inconsistencies in symptom disclosure arising from the interplay of sex‐ and sport‐specific sociocultural factors, along with individual personality traits (Dick, 2009; Kroshus et al., 2017). Neuroimaging studies that combine standard clinical protocols with assessments of attitudes surrounding symptom reporting are needed to resolve these questions.

A significant main effect of concussion on CBF was observed at all imaging sessions, with reduced CBF in frontal, temporal, and parietal regions. The findings are consistent with evidence that frontotemporal regions are vulnerable to primary injury (Tarlochan, 2013) and replicate previous studies of sport‐related concussion that reported decreased frontotemporal CBF years after the concussion event (Churchill et al., 2017; Churchill et al., 2019a). Sex‐specific effects were also identified, with male athletes having greater reductions in occipital‐parietal CBF than female athletes, at all imaging sessions. These findings align with a previous study that examined HOC effects for a subset of controls used in the present work (Hamer & Churchill, 2019) and reported significantly reduced CBF for males but not for females. In the present study, CBF declines are detectable among recently concussed male athletes, even after adjusting for these HOC effects. The acute CBF declines may be driven by multiple factors, including microvascular injury, global neurometabolic dysregulation, and injury to neural mechanisms involved in blood flow regulation (Benarroch, 1993; Len & Neary, 2011; Werner & Engelhard, 2007). The CBF response also persists at RTP and 1‐year post‐RTP, with comparable effect sizes throughout. Long‐term alterations in CBF may reflect the evolution of acute injury mechanisms, for example, microvascular injury may lead to neuroinflammatory response (Gurney, Estrada, & Rosenberg, 2006; Truettner, Alonso, & Dietrich, 2005) that further impairs vascular integrity (Sweeney, Zhao, Montagne, Nelson, & Zlokovic, 2019), and the injury of regions implicated in CBF regulation may lead to axonal degeneration (Dikranian et al., 2008; Spain et al., 2010). Over the longer term, subtle GM volume loss may also contribute to these effects by reducing regional CBF demand (Churchill et al., 2017).

The sex differences in CBF response may be explained in part by the neuroprotective effects of female sex hormones (Wagner et al., 2004). Following a traumatic injury, estrogen has been shown to help maintain cerebral autoregulation (Roof & Hall, 2000) and to mitigate the excitotoxic effects of glutamate in the brain (Mendelowitsch, Ritz, Ros, Langemann, & Gratzl, 2001), both of which may protect against post‐concussion disruptions in CBF. Another potential contributing factor is the greater reactivity of the female cerebrovascular system (Kastrup et al., 1997; Tallon et al., 2020). The present results suggest that, despite females having higher basal metabolism and resting CBF (Rodriguez, Warkentin, Risberg, & Rosadini, 1988), the increased reactivity may help to more effectively buffer the effects of concussion on CBF during recovery. Conversely, CBF reductions in male athletes may be exacerbated by higher rates of subconcussive impacts typically seen in this cohort (Saunders, Le, Breedlove, Bradney, & Bowman, 2020). Subconcussive blows are associated with increased vascular permeability (Marchi et al., 2013), potentially sensitizing athletes to CBF perturbations after a concussion. The localization of sex differences to occipital‐parietal regions is also noteworthy. It is presently unclear whether this truly represents an area of greater relative vulnerability among male athletes, or whether sex differences are more readily detected in this area due to high vascular density, which enhances the ASL signal. This is important to investigate further, as impaired function in these regions, which are involved in visuospatial processing, attention, and memory (Kravitz, Saleem, Baker, & Mishkin, 2011), may put an athlete at greater risk for reinjury.

No main effects of concussion on FA were identified. The absence of significant findings differs from previous studies of sport‐related concussion, where reduced FA was seen at acute injury and RTP but had resolved 1 year afterward (Churchill et al., 2017b; Churchill et al., 2019a). The lack of significant findings suggests that concussion effects on FA are heterogeneous between athletes. This is supported by prior literature, as the FA response varies from study to study, with reports of FA decreases, increases, and/or a combination of both (Eierud et al., 2014). One source of variability appears to be sex differences, as this study identified elevated FA among concussed male athletes but reduced FA among concussed female athletes. Decreased FA, as seen in the female cohort, is frequently reported after mild TBI (Shenton et al., 2012) and may represent a combination of vasogenic and cytotoxic edema (Donkin & Vink, 2010), along with inflammatory‐mediated glial activation and diffuse WM injury (Inglese et al., 2005; Streit, Mrak, & Griffin, 2004), with the latter processes likely contributing more to the post‐acute declines in FA seen at RTP and 1‐year post‐RTP. In general, lower FA values after a TBI have been linked to WM injury and worse long‐term clinical outcomes (Kumar et al., 2009; Sidaros et al., 2008).

By contrast, less is known about the mechanisms of elevated FA, as seen in the male cohort. Earlier studies of concussed athletes attributed elevated FA to edema, altered myelin water content, and glial activation (Bazarian et al., 2007; Wilde et al., 2008). However, in recent studies, elevated FA was correlated with better long‐term cognitive and functional outcomes (Strauss et al., 2016; Yin et al., 2019). Additionally, animal models suggest that long‐term increases in FA are unlikely to be caused by gliosis and may instead reflect structural reorganization (Harris, Verley, Gutman, & Sutton, 2016). Our findings differ from a previous study which found greater FA reductions in males than females (Fakhran et al., 2014), albeit for nonathletes and for a wider age range (10–38 years), with males having longer recovery than females. In the sport domain, findings are more aligned with a study of subconcussive impacts in hockey players (Sollmann et al., 2018), where females had reduced FA but not males. Given the literature evidence, FA results may be tentatively interpreted as a sign of greater diffuse injury in females, potentially due to differences in concussion biomechanics. This may include proportionately reduced head/neck musculature giving females a reduced ability to buffer concussive blows (Tierney, 2004), with correspondingly greater WM effects. The observed FA effects were primarily in the corona radiata, which is a frequently identified region in studies of concussion and mild TBI (Eierud et al., 2014). However, the FA results should be interpreted with caution until validated using more sophisticated models of tissue microstructure, such as myelin water imaging and multishell diffusion‐weighted techniques (Laule et al., 2004; Zhang, Schneider, Wheeler‐Kingshott, & Alexander, 2012).

An extensive main effect of concussion on MD was observed at all imaging sessions, with effects mainly in the superior corona radiata. This replicates previous studies of sport‐related concussion, which reported elevations in MD beyond RTP (Churchill et al., 2017b; Churchill, Hutchison, Graham, & Schweizer, 2019b), and is consistent with the hypothesis that MD is more sensitive to concussion than FA (Cubon, Putukian, Boyer, & Dettwiler, 2011). Similar to FA, the elevations in MD are associated with acute edema, with subsequent glial activation and diffuse WM injury also contributing (Donkin & Vink, 2010; Streit et al., 2004). The analysis of sex differences identified different effects compared to FA, with concussed male athletes having greater MD elevations in the corpus callosum and posterior thalamic radiation compared to female athletes, at all imaging sessions. The observed differences between FA and MD are consistent with literature showing different spatial patterns of effect for different DTI parameters after a concussion (Cubon et al., 2011).

Comparing with prior literature, our findings for MD differ from a study of sub‐concussive impacts over a season of ice hockey (Sollmann et al., 2018), where females had elevated diffusivity but not males. This suggests distinct microstructural processes occurring during concussion recovery, compared to repeated exposure to sub‐concussive blows. The presence of effects in the corpus callosum is consistent with biomechanical models of injury which predict strong shear forces in this region (Viano et al., 2005), hence this may again reflect differences in male and female injury biomechanics, although sex differences in glial response may also play a role (Caplan, Cox, & Bedi, 2017). Interestingly, male athletes exhibited greater disturbances in both CBF and MD values compared to female athletes, with the greatest effects at 1‐year post‐RTP, suggesting the MRI parameters may be interrelated. For example, higher MD in the corpus callosum may correlate with disrupted interhemispheric communication of networks involved in CBF regulation (Benarroch, 1993). This hypothesis should be further investigated in future research that targets these networks and potentially integrates peripheral indices of autonomic function (Napadow et al., 2008).

Although this study provided new insights into sex differences in concussion recovery, there were also some limitations that should be acknowledged. One such issue surrounds cohort matching. The present study used a normative approach that adjusted for effects of age, sex, and presence of HOC on MRI parameters. This strategy was chosen to maximize the robustness and stability of estimates, focusing on key demographic factors that have a well‐established impact on brain physiology. Nevertheless, other uncontrolled demographic factors may contribute to modeling error. This may include HOC features, such as total number of concussions and time since the last injury, along with unmeasured aspects, such as clinical outcomes of the previous injury. There are also considerations of sport representation; the present study used a mixed sample of athletes drawn from different sports, focusing on effects that generalize across sports. However, cohort matching based on sport, position played, and even playing style may improve the precision and accuracy of MRI parameter estimates. Similarly, future research should monitor and adjust for the effects of contact exposure, to determine whether sex differences in subconcussive impacts play a role in the observed imaging effects. Lastly, there was some participant drop‐out at all imaging sessions, with greatest attrition at 1‐year post‐RTP. Based on correlation testing, the dropout did not have evident demographic or clinical biases, but other unmodeled sources of attrition may exist. These issues highlight the need for further longitudinal studies of concussion examining both clinical and imaging data, to determine the underlying factors contributing to sex differences in neuroimaging measures.

Overall, this study provides evidence of acute and long‐term sex differences in the neurobiological response to sport concussion. Although the physiological disturbances lasted beyond RTP for both cohorts, all athletes had returned to normal activities without persistent cognitive or symptom complaints at 1‐year post‐RTP. Nevertheless, based on evidence of worse outcomes if a second concussion occurs before recovery is complete (McLendon, Kralik, Grayson, & Golomb, 2016), future research should examine longer post‐concussion time intervals and athletes with multiple concussions. It is presently unclear if and when the perturbations in CBF and WM normalize, although HOC studies show significant long‐term effects (Churchill et al., 2017; Hamer & Churchill, 2019; Inglese et al., 2005), suggesting that such a process may span multiple years after injury. Given the hypothesized protective effects of female sex hormones on CBF, it will be important for future work to account for menstrual phase and use of oral contraceptives within female cohorts. Similarly, given the hypothesized relationship between sex differences in WM and injury biomechanics, future work should incorporate measures of dynamics (e.g., telemetry) and kinematics (e.g., head–neck stiffness and strength) when assessing WM injury. If validated in future research, these findings also point towards sex‐specific interventions, such as hormone treatments among male athletes, and training for neck strength and flexibility among female athletes. Given the persistent nature of sex‐specific brain changes after concussion, such interventions may have long‐lasting effects on brain physiology.

## CONFLICT OF INTEREST

The authors declare no potential conflict of interest.

## ETHICS STATEMENT

The study was in accordance with the Canadian Tri‐Council Policy Statement 2 and approval of the University of Toronto and St. Michael's Hospital research ethics boards.

## CONSENT TO PARTICIPATE

All participants gave free and written informed consent.

## CONSENT FOR PUBLICATION

All manuscript authors have provided consent to publish.

## CODE AVAILABILITY

All software will be shared by request from any qualified investigator.

## Supporting information


**Appendix** S1: Supporting information

## Data Availability

The authors have documented all data, methods, and materials used to conduct this research study, and anonymized data will be shared by request from any qualified investigator.
