# Supplementary material for: Sex differences in acute and long‐term brain recovery after concussion
Source: Hum Brain Mapp. 2021 Oct 12;42(18):5814–26. doi: 10.1002/hbm.25591 (PMC8596946; doi:10.1002/hbm.25591)
Supplement: Supplementary file 1 — Appendix S1: Supporting information [file HBM-42-5814-s001.docx]

**Appendix-1:** Multivariate outlier detection

To identify outlying imaging data, we used a simple protocol that quantified overall deviation of brain maps from a robust mean map. For a parameter map of interest (cerebral blood flow (CBF), fractional anisotropy (FA), mean diffusivity (MD)), the data of subject *s* = 1…*S* are formed into vectors $\boldsymbol{x}_{s}$ consisting of *V* measurements. All subject data are then concatenated into a 2D matrix $\boldsymbol{X}$ of dimensions (*V* x *S*). We then compute map of median values $\boldsymbol{x}_{med}$ over all subjects, then obtain for each individual the sum-of-squares deviation $SSD\left( s \right)=\sum_{v=1}^{V} \left( \boldsymbol{x}_{s}\boldsymbol{-}\boldsymbol{x}_{med} \right)^{2}$. We then fit a gamma distribution to the set of SSD values using maximum likelihood estimation, and subsequently obtain p-values as the reciprocal of the cumulative probability for each participant’s SSD. Finally, we identify all datapoints having significant SSD values at a False Discovery Rate (FDR) threshold of 0.05.

Figure S1 below shows an example plot from the CBF data, where each vector $\boldsymbol{x}_{s}$ corresponds to the set of 38,638 voxel values. For this parameter, N=6 scans showed abnormal values at an FDR of 0.05 (2 control scans, 2 RTP scans, 2 1YR scans). For DTI measures of FA and MD, $\boldsymbol{x}_{s}$ corresponds to the set of 16,541 white matter voxels. For FA, we identified N=2 abnormal scans, and for MD, we identified N=2 abnormal scans.


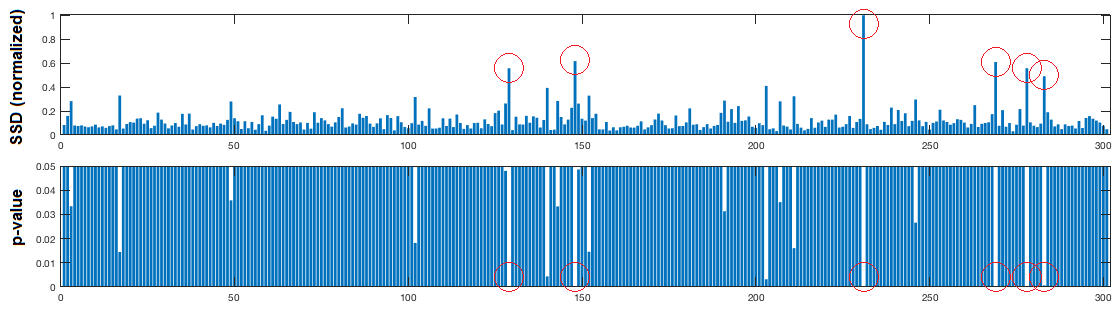


**Figure S1**: plot depicting outlier estimation, including (top panel) sum-of-squares deviations (SSD) from the median brain map, with values rescaled to the range of [0, 1]; and (bottom panel) corresponding p-values, focusing on the interval of [0, 0.05].
